# Recognizing Debilitating and Fatal Dysautonomia in Patients With Parkinson’s Disease in the Emergency Room: A Case Report and Narrative Review

**DOI:** 10.7759/cureus.65383

**Published:** 2024-07-25

**Authors:** Chaoneng Wu, Kashiff Ariff, Sujata Kambhatla, Barry Brenner

**Affiliations:** 1 Internal Medicine, Garden City Hospital, Garden City, USA; 2 Neurology, Garden City Hospital, Garden City, USA; 3 Emergency Medicine, Garden City Hospital, Garden City, USA

**Keywords:** orthostasis, constipation, neurogenic bladder, dysphagia, gastroparesis, parkinson’s disease, dysautonomia

## Abstract

Dysautonomia impacts multiple systems leading to a spectrum of severe disorders independent of the motor symptoms in Parkinson’s disease (PD). Although the motor symptoms of dyskinesia and immobility in patients with PD were traditionally considered the major reasons leading to emergency visits, the significance of non-motor symptoms, particularly dysautonomia-related disorders, have been increasingly appreciated during their emergent encounters. We present the case of an elderly patient with advanced PD who was hit by a full spectrum of dysautonomia-related disorders, had frequent emergency visits and hospital admissions over one year, and eventually died on his fifth emergency visit. His dysautonomia-related disorders included dysphagia, gastroesophageal reflux disease, neurogenic bladder, chronic constipation, and cardiac dysautonomia with orthostatic intolerance. We further review emergent presentations, assessments, and immediate management of these dysautonomia-associated disorders in patients with PD. In summary, these dysautonomia-linked comorbidities can be debilitating and sometimes fatal. As for our case, the patient was on a clinical decline majorly due to dysautonomia and nearing the end of life over the past year. A holistic approach of possible de-escalating care and palliative care might lead to a better quality of life for the patients and their families. Nevertheless, generally speaking, emergent presentations of dysautonomia symptoms in patients with PD should be recognized and treated timely and appropriately in the emergency room. Emergency clinicians need to increase awareness and make efforts to manage these acute worsening episodes of dysautonomia disorders in patients with PD to prevent debilitating and fatal complications.

## Introduction

Parkinson’s disease (PD) has become one of the most common neurodegenerative disorders with increasing global prevalence. In North America, according to the 2016 Global Burden of Disease study, there were an estimated 930,000 PD patients in 2020, with an estimation of 1,238,000 PD patients by 2030 with a typical onset age of 60 to 70 years [1]. Over one-third of these elderly PD patients had at least one emergency department (ED) visit or were hospitalized once annually. Accordingly, the annual economic cost of PD exceeds $23 billion in the United States, leading to significant financial burdens and social pressures [1].

PD was traditionally considered a motor disorder characterized by bradykinesia, resting tremor, rigidity, and postural instability. Severe motor “off” periods including dyskinesia, immobility, and psychosis were reported to be the primary presentations during emergency visits [2]. However, the importance of non-motor symptoms, particularly dysautonomia-related disorders or comorbidities, has been increasingly recognized over the past few years. For example, aspiration pneumonia due to pharyngeal or esophageal dysautonomia constitutes the most common reason for emergency admission of PD patients [3]. Another study revealed that falls with subsequent fractures or contusions were the major reason for emergency visits and admissions in PD patients, followed by comorbidities such as cardiovascular emergencies, infections, abdominal pain, and altered mental status [4]. Dysautonomia is closely related to the majority of these comorbidities.

Dysautonomia in PD patients involves a broad clinical spectrum of disorders. Cardiovascular dysfunction is characterized by orthostatic hypotension (OH), supine hypertension, or postprandial hypotension. The gastrointestinal (GI) dysfunction involves all levels of the GI tract. The urinary dysfunction entails difficulty voiding to too frequent voiding. The altered sweating and thermoregulatory abnormalities are usually present but not widely recognized. Some of these symptoms can be debilitating and even lead to fatal complications in affected patients. Nevertheless, these problems are constantly underestimated in emergency practice. Recognizing and managing dysautonomia-related disorders, especially acute worsening episodes, can reduce their footprint on the emergency healthcare system.

## Case presentation

**Figure 1:**
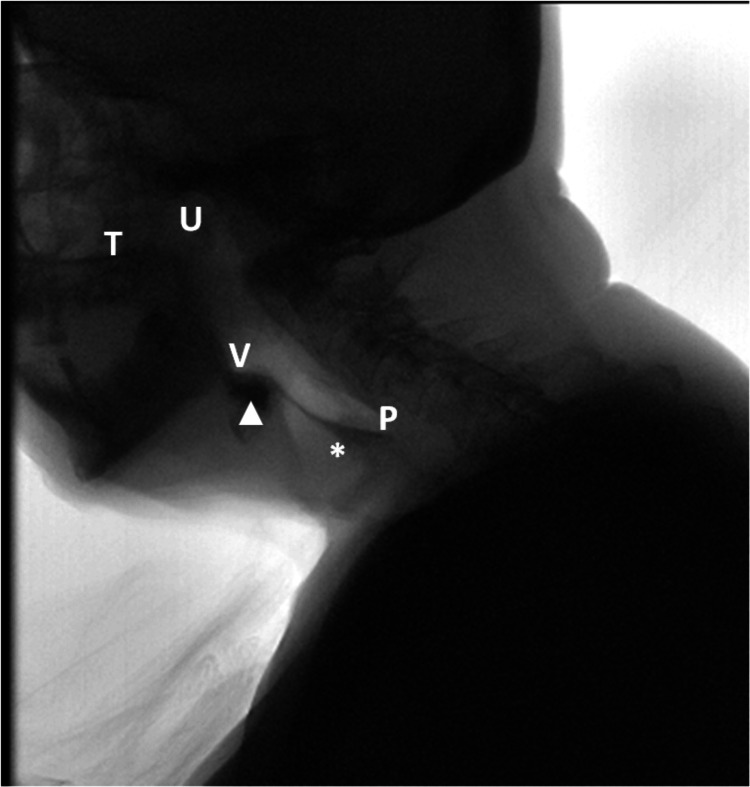
Videofluoroscopic swallowing study revealing oropharyngeal dysphagia. The lateral fluoroscopic view of the pharynx demonstrates moderate impairment of the pharyngeal and oral phases of deglutition. There is residue within the vallecular (white triangle) and pharyngoesophageal segment (white star). Structures seen include the soft palate (U), the tongue (T), the valleculae (V), the epiglottis (E), and the piriform sinuses (P).

An 85-year-old man was brought to the ED for intractable vomiting of coffee-ground emesis for two hours. He was diagnosed with PD 20 years ago and stopped all medications six months ago due to recurrent aspirations. He had three recent admissions for recurrent aspiration due to oropharyngeal dysphagia as shown by an abnormal videofluoroscopic swallowing study (VFSS) (Figure 1). He had a percutaneous endoscopic gastrostomy (PEG) tube placed for nutritional support eight weeks before this ED visit. After the PEG tube placement, he refused to restart his medications.

**Figure 2:**
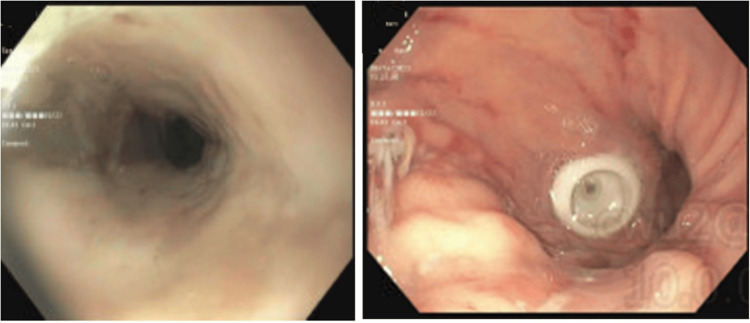
Esophagogastroduodenoscopy showing erosive esophagitis and hemorrhagic gastritis. Left: Esophagitis with no bleeding. Right: An erosion with stigmata of recent bleeding in the gastric body and a patent G-tube.

On assessment, the patient had a respiratory rate of 31 breaths/minute and oxygen saturation of 85% on room air. The temperature was 35.8°C, blood pressure was 85/54 mmHg, and heart rate was 121 beats/minute. He appeared pale and was unresponsive with a Glasgow Coma Scale score of 8. He was intubated immediately. An orogastric tube was placed due to skin irritation around the gastrostomy site. Subsequently, 250 mL of coffee-ground drainage was observed immediately. The hemoglobin was 6.7 g/dL and the white blood cell count was 23 × 10^9^/L. He was diagnosed with acute hemorrhagic anemia and shock. When receiving fluid resuscitation (30 mL/kg), one unit of packed red blood cells, and proton pump inhibitor infusion, intensive care unit (ICU) admission was initiated. In the ICU, esophagogastroduodenoscopy (EGD) showed erosive esophagitis and hemorrhagic gastritis (Figure 2).

**Figure 3:**
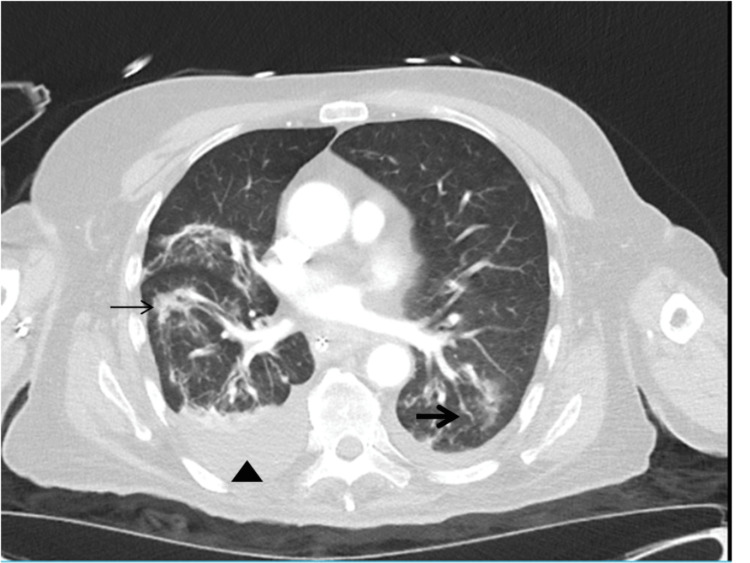
CT scan showing multilobular lung infiltration. CT chest scan showing diffuse, patchy, ground-glass, and nodular pneumonic infiltrates in the right lung in the upper middle and lower lobes (thin arrow), and mostly lower lobe, patchy, ground-glass, nodular infiltrates with consolidation in the left lower lobe (thick arrow). There is a small right pleural effusion (triangle).

He developed hypoxemic respiratory failure secondary to aspiration pneumonia (Figure 3).

**Figure 4:**
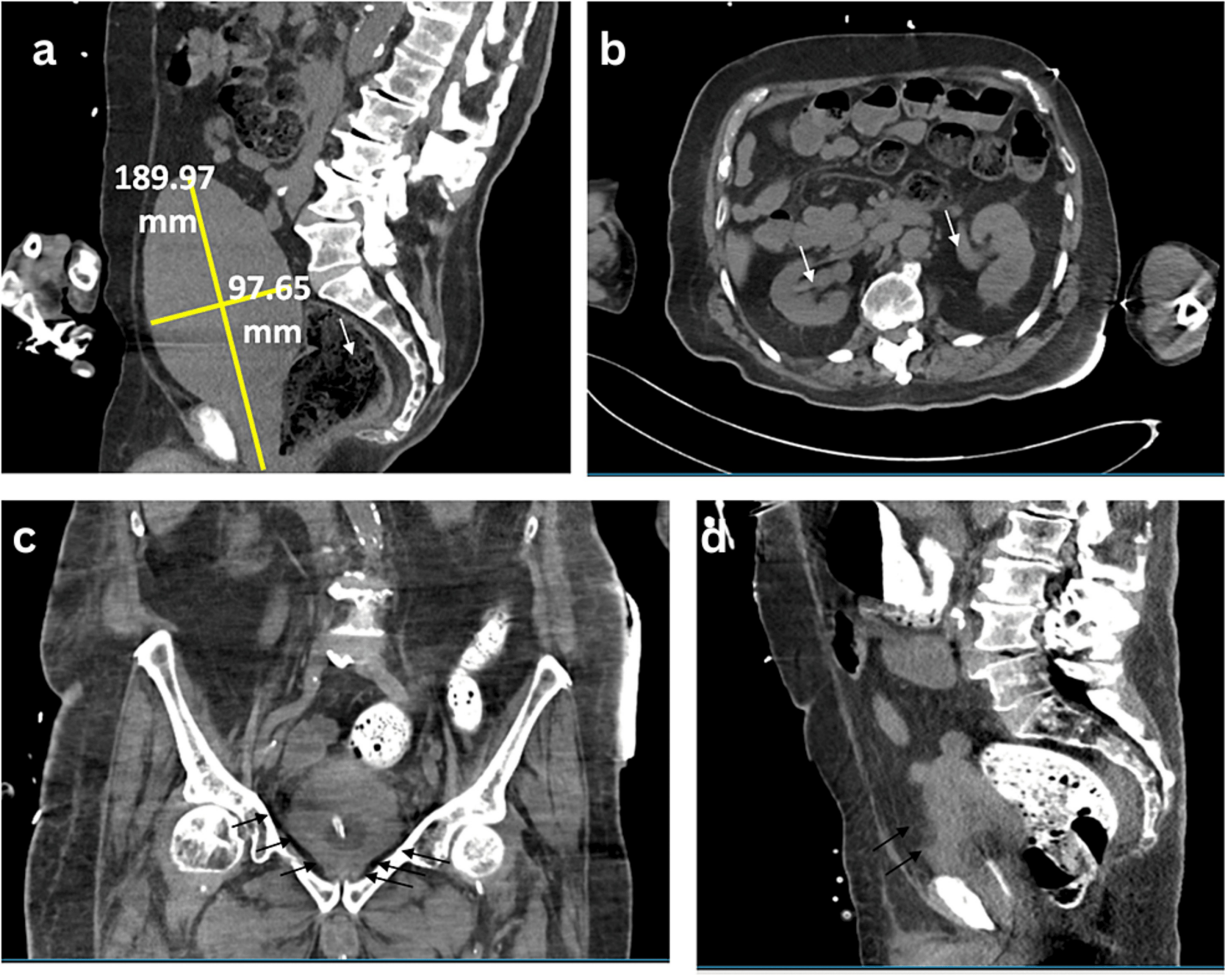
CT pelvic scan showing distended and conical configuration bladder with emphysema cystitis and stool impaction. a: The bladder appears distended with a conical configuration. There is funneling of the bladder neck with mild bilateral hydronephrosis. b: Bilateral hydronephrosis (white arrow). It suggests a neurogenic bladder and bladder outlet obstruction with obstructive nephropathy. The lower panels of the CT pelvic scan (c and d) show emphysema cystitis with markedly abnormal bladder. The thickening bladder wall contains gas (black arrows), suggesting emphysematous cystitis (bottom left, coronal view; bottom right, sagittal view).

The sputum and blood culture from tracheal suction grew extended-spectrum beta-lactamases (ESBL)-positive *Escherichia coli*. He had neurogenic bladder, obstructive nephropathy, and hydronephrosis (Figures 4a, 4b) with urinary tract infection (UTI) and emphysematous cystitis (Figures 4c, 4d) as urine culture showed ESBL-positive *Klebsiella oxytoca* with multiple drug resistance.

He received meropenem treatment. He had stool impaction and colonic bowel obstruction on an abdominal CT scan and received manual disimpaction, polyethylene glycol, and senna-docusate. He was gradually weaned off mechanical ventilation and vasopressors and improved enough to be discharged to a rehabilitation facility. Before his discharge, he experienced OH. His orthostatic vital signs showed lying-down blood pressure of 115/70 mmHg and heart rate of 85 beats/minute; sitting-up blood pressure of 102/65 and heart rate of 86 beats/minute; and standing-up blood pressure of 90/60 and heart rate of 88 beats/minute. While standing up, he experienced lightheadedness, but otherwise, no palpitation, chest pain, shortness of breath, or diaphoresis was reported. He was prescribed midodrine. Three weeks later, he was brought back to the ED due to another episode of upper gastrointestinal bleeding (UGIB). He expired from a catastrophic aspiration and cardiopulmonary arrest.

## Discussion

The current case discussed a wide spectrum of manifestations of autonomic dysfunction. The patient’s GI pathogenesis involved the oropharyngeal and esophagogastric motility disorders (EMDs) and colonic motility disorders. He also had neurogenic lower urinary tract dysfunction (NLUTD) with a neurogenic bladder. His cardiovascular symptoms included OH. This broad clinical spectrum marks the important features of dysautonomia in PD patients requiring emergent recognition and interventions.

Dysphagia and esophagogastric motility disorders

Dysphagia involves pharyngeal and/or esophageal dysfunction with difficulty in initiating and/or completing swallowing. EMD represents abnormal coordination of esophageal peristalsis and relaxation of the sphincters. Dysphagia occurs in a relatively late stage of PD but can also be an early presentation. A study showed that 50% of PD patients reported symptomatic dysphagia, but with a barium swallowing study or VFSS, the occurrence of dysphagia increased from 75% to 97% [5]. Dysphagia can lead to dehydration, malnutrition, and impaired medication intake. As the major mortality risk, dysphagia-associated aspiration pneumonia with severe respiratory complications accounted for 25% of mortality, the leading cause of death among patients with PD [6].

Clinically, dysphagia in PD typically occurs with both solid and liquid food, while dysphagia with mechanical esophageal obstruction occurs only with solid food. Although motor dysfunction impairing the muscles of the face and mouth are related to dysphagia, issues impacting the throat and swallowing are typically dysautonomia. Accordingly, repetitive pump movements of the tongue, premature spillage, and oral residue indicate dysphagia occurring during the oral phase of swallowing, whereas retaining residue in valleculae or pyriform sinuses and decreased rate of spontaneous swallowing implies pharyngeal dysphagia [7]. The esophageal dysphagia has the pattern of esophageal spasm, slow esophageal transit, aperistalsis, gastroesophageal reflux, and the motor disorders of the lower esophageal sphincter [7]. Regurgitation of previously swallowed but undigested food can be a typical sign of advanced achalasia. Patients with severe chest pain and dysphagia can also mimic cardiac ischemia. In addition to dysphagia, the long-standing esophageal achalasia and gastroesophageal reflux can lead to erosive esophagitis or stasis ulcers, which can further cause UGIB with even hemorrhagic shock, as seen in our case.

Screening for dysphagia in patients with PD starts with simple bedside swallowing tests by the nursing staff or more elaborate multi-consistency protocols by speech and language therapists (SLTs). Flexible endoscopic evaluation of swallowing (FEES) is a portable tool that can be performed by an emergency physician or SLTs via using a flexible endoscope passing into the hypopharynx to observe the pharyngeal swallowing motion directly [7]. VFSS, also known as modified barium swallow, is performed usually as an outpatient. According to the Clinical Practice Guidelines for Oropharyngeal Dysphagia [8], early VFSS is highly recommended for screening and assessment of dysphagia. In our case, the dysphagia was confirmed by FEES performed as an inpatient by SLTs. Currently, the diagnosis and management of dysphagia have been relegated mostly to SLTs. The lack of specific training in diagnosing and managing dysphagia is a problem with emergency physicians. More research is needed to generate evidence-based clinical practice to confidently diagnose and treat PD-associated dysphagia starting in the ED.

Gastroparesis

Gastroparesis is defined as delayed gastric emptying without obstruction. The prevalence of gastroparesis in PD ranges from 70% to 100% [5]. Pathologically, PD alters the motor activities of various stomach segments mediated by dysfunctional vasovagal neural circuits via smooth muscle and interstitial cells of Cajal. The typical presentations in the ED would be progressive nausea, abdominal distention, and cramp pain relieved by vomiting old food residue. The abdominal pain is usually described as vague, burning, or cramping located at the epigastrium, whereas sharp and well-localized pain prompts the clinicians to rule out other causes. Physical examination reveals signs of sunken eyes, dry mucous membranes, poor skin turgor, and orthostatic intolerance, which should mandate prompt fluid resuscitation. Patients may develop abdominal distention and tympany without tenderness. One may elicit a succussion splash by rocking the patient from side to side gently. Consequently, it leads to malnutrition, weight loss, and dehydration, associated with hypokalemia and hypochloremic metabolic alkalosis. It also delays levodopa absorption and exacerbates motor dysfunction. Abdominal radiographs, especially CT scans, are useful to rule out entities such as gastric outlet obstruction or small bowel obstruction. It also gives additional details as the thickness of the gastric wall and pylorus. Endoscopy is needed to rule out mechanical obstruction. Gastric emptying scintigraphy as a gold-standard study for gastroparesis is usually performed as an inpatient. A nasogastric tube can be inserted with suction for decompression and reduction of the risk of aspiration. Generally, an ED evaluation identifies the associated complications and severity of gastroparesis rather than making the definitive diagnosis of gastroparesis.

Treatment for gastroparesis includes altering the meal frequency and content. Patients should be encouraged to eat more liquid-based meals and less non-digestible (insoluble) fibers to facilitate gastric emptying, such as whole-wheat flour, nuts, beans, and vegetables such as potatoes and cauliflower. Domperidone is a peripheral dopaminergic blocking agent with little or no effect on central dopamine receptors and has antiemetic and prokinetic effects. It has been documented to be a common and safe clinical approach for antiemesis. Although it is not available in the United States, it can be easily obtained from Canada and is not costly.

Constipation

Constipation has seen a significant increase in ED visits from 2006 to 2017 based on a nationwide, cross-sectional study of ED encounters [9]. Constipation presents in up to 80% to 90% of patients with PD. Pathologically, reduced myenteric ganglion with an accumulation of phosphorylated α-synuclein in the autonomic nerve plexus causes dysautonomia, which can be further exacerbated by the adverse effect of anti-PD drugs, especially anticholinergics, and dopamine agonists due to delays in colonic transit. The major complaints include delayed passage of stools (fewer than two passages weekly), hard stools, a sensation of incomplete evacuation, or the need for excessive straining or longer time spent on the toilet. Constipation Rome criteria II is a useful scale to initially assess constipation.

The symptoms associated with constipation in PD can be severe, debilitating, and even life-threatening. Our patient developed constipation complicated with impaction necessitating manual disimpaction of the impacted stool. During ED encounters, it is crucial to include broad differentials of concomitant diseases before committing to the diagnosis of constipation, such as diverticulitis, incomplete evacuation and bowel incontinence, megacolon, colon obstruction, pseudo-obstruction, volvulus, and perforation. Accordingly, constipation in PD-related ED visits requires imaging studies, such as a CT scan of the abdomen and pelvis. Digital rectal examination (DRE) is useful to differentiate constipation due to slow colonic transit versus dyssynergic defecation, a condition caused by an inability to relax pelvic floor muscles during defecation because of the impaired dopaminergic motor disorder. DRE can also identify structural abnormalities, such as tenderness, mass, stricture or spasm, or fecal impaction [10]. One should also compare the resting and squeezing anal sphincter tones [10]. Emergency intestinal pseudo-obstruction (IPO) occurs in about 2.4% of patients with PD with images showing bowel dilation but no anatomical obstruction [11]. It has the symptoms of abdominal pain, distension, nausea, vomiting, and obstipation. IPO requires hospitalization and has poor outcomes. Paradoxical diarrhea can present in patients with PD where watery stool leaks around a hard stool mass out of the anus. The differentials may include bacterial infection, malignancy, or bowel compression from urinary retention.

Currently, clinical trials in evaluating the efficacy of various drugs for constipation in PD remain scarce. One can first start with osmotic laxatives, represented by polyethylene glycol. Macrogol (laxative) improves bowel movement frequency and stool consistency. Then, stimulant laxatives serve as rescue options, such as bisacodyl, senna, and sodium picosulfate. Other methods include a fiber-rich diet, exercise, and liquid intake, along with psyllium as a bulk laxative. Methylnaltrexone can be effective in managing opioid-induced constipation as PD patients may receive chronic opioids or opioid-like medications for pain. Modern prokinetic agents such as serotonin (5-HT4) agonists and new drugs of chloride channel activators such as linaclotide and lubiprostone are still controversial and may be useful in PD-related constipation.

Neurogenic lower urinary tract dysfunction

Dysautonomia impacts the micturition reflex, the detrusor muscle, and the sphincter of the bladder and urethra, leading to NLUTD, a term previously described as “neurogenic bladder” [12]. The vast majority of PD patients experience NLUTD independent of their motor functions. Common manifestations of NLUTD in PD patients may include urge incontinence, nocturia, and overactive bladder, meaning a frequent and urgent desire to urinate even though the bladder is not full. It has been reported that approximately 80% of PD patients experience bladder problems, yet only 15% of individuals develop troublesome incontinence [13]. The initial urologic evaluation in the ED includes standard history, frequency-volume queries, physical examination and urinalysis and culture, ultrasonography or CT scan, or post-void residual (PVR) as indicated. Non-neurogenic lower urinary tract symptoms may mimic or occur concomitantly with NLUTD, such as urological stones, UTIs, or malignancies [14]. Some other concomitant conditions include obstructive voiding symptoms due to an enlarged prostate in a male patient, or a female PD patient with symptoms of stress urinary incontinence. Many PD patients may also have diabetic neuropathy, lumbar spondylosis, or incomplete emptying secondary to a cerebrovascular accident, which complicates bladder symptoms [15]. In our patient, the CT scan showed a distended and conical configuration with bilateral hydronephrosis, which is consistent with NLUTD with obstructive nephropathy requiring a Foley catheter to decompress the bladder. He further developed emphysema cystitis with a multidrug-resistant infection.

Patients with PD developing NLUTD result in urinary retention being prone to complicated UTI with a risk of antimicrobial resistance and the possible need for catheterization to decompress the bladder. A study showed that almost 40% of NLUTD developed a UTI over a one-year period [12]. Catheterization to manage the bladder with disturbed hydrokinetics, elevated PVR, and vesicoureteral reflux increases the risk of UTI. These patients may not present typical alarming signs of flank or abdominal pain due to the altered sensation from the PD. Fever or not responding to appropriate therapy remains a warning sign of upper urinary tract infection for sources of UTI, including pyonephrosis, hydronephrosis, urolithiasis, or renal abscess. Imaging is essential to examine both upper and lower urinary tracts. Aseptic urine specimens via the urethral or suprapubic catheter port may be vital for a correct diagnosis and treatment.

As UTI is a leading cause of acute neurological deterioration in patients with PD, there is a low threshold to treat dysautonomia-associated UTI, preferably referring to other recent available culture results. Usually, urology will make the recommendations regarding intermittent catheterization over indwelling catheters to facilitate bladder emptying, and suprapubic catheterization over an indwelling catheter upon requiring a chronic indwelling catheter. Oral antibiotic prophylaxis should only be considered in highly selected patients with frequently recurrent UTIs, unsuccessful behavioral modification, and non-antimicrobial treatment [16].

Cardiovascular dysautonomia

Cardiovascular dysautonomia is commonly observed in approximately 80% of PD patients. It includes a wide spectrum of disorders such as OH, supine hypertension, labile blood pressure, absence of blood pressure decrease during the night (non-dipping), abnormal circadian blood pressure patterns, and prolonged corrected QT interval. OH is the most common manifestation with a varied prevalence from 10% to 65% [17]. The underlying pathology is dysregulation of sympathetic noradrenergic innervation of the cardiovascular system and baroreflex failure with decreased serum catecholamine levels [18].

These patients present with dizziness, lightheadedness, general weakness, exercise intolerance, or fainting or syncope while standing. They usually have labile blood pressure without compensatory tachycardia. These symptoms can lead to falls and cognitive decline. They also increase the risk of developing ischemic heart disease, cardiac arrhythmias, and heart failure in patients with PD. By definition, OH means a sustained drop in systolic blood pressure ≥20 mmHg and/or diastolic blood pressure ≥10 mmHg in three minutes of standing [19]. Standing systolic blood pressure <90 mmHg is highly suggestive of orthostasis predicting symptoms of orthostatic intolerance. If blood pressure falls mildly in the third minute, the recommendation is to prolong the orthostatic challenge to 5-10 minutes for delayed orthostasis [19].

Non-neurogenic causes as well as exacerbating factors for OH must be evaluated, such as infections, occult hemorrhage, anemia, dehydration, and polypharmacy, including antihypertensive agents, opioids, α-blockers, tricyclics, and neuroleptics. Dopaminergic drugs can result in OH, such as levodopa-carbidopa. There are two approaches to distinguish neurogenic from non-neurogenic orthostasis. First, due to an insufficient baroreflex in neurogenic orthostasis, only small or no orthostatic heart rate rise is seen with blood pressure dropping upon standing, which would be markedly increased to counteract the blood pressure drop in non-neurogenic orthostasis. Accordingly, an increase in heart rate <0.5 beats/minutes/mmHg of systolic blood pressure fall upon the three-minute tilt table test suggests neurogenic orthostasis. Second, with a Valsalva maneuver, patients with neurogenic orthostasis will not have the physiological blood pressure increase upon finishing the maneuver, which indicates an insufficient noradrenergic stimulation to the receptive blood vessels [20]. Our patient developed OH and met the diagnostic criteria of dropping blood pressure to ≥20/10 mmHg while feeling lightheadedness. An outpatient tilt-table study would be more accurate to confirm the diagnosis and differentiate neurogenic from non-neurogenic orthostasis.

Patients should be educated to avoid triggering factors of OH, such as a sudden body position change, alcohol, strenuous activity, and exposure to hot environments. Increasing water and salt intake and adjustment of drugs that worsen OH, such as dopamine agonists, monoamine oxidase-B inhibitors, and antihypertensive medications, may be crucial to prevent falls. The commonly recommended medications for OH are midodrine, droxidopa, and hydrocortisone [19].

Heart rate variability (HRV) is another indicator of the tone of the cardiac autonomic nervous system, particularly parasympathetic modulation, which refers to the fluctuating time between successive heartbeats [21]. Clinicians and researchers assess HRV using time-domain, frequency-domain, and non-linear indices. There are commercial apps to track HRV. Nevertheless, currently, there is no recommended protocol, concurrent validity criteria, or normative values for HRV analysis in PD patients. Resting tachycardia with chest pain is uncommon in PD with dysautonomia. Clinicians should perform atherosclerotic checkups and cardiovascular imaging workups.

Common autonomic dysfunctional manifestations in patients with PD in the emergency room are listed in Table 1.

**Table 1:** Common dysautonomic manifestations in patients with PD in the emergency room. GI: gastrointestinal; GU: genitourinary; CV: cardiovascular; EMD: esophagogastric motility disorder; VFSS: videofluoroscopic swallow study; FEES: flexible endoscopic evaluation of swallowing; EGD: esophagogastroduodenoscopy; DRE: digital rectal examination; CT: computed tomography; NLUTD: neurogenic lower urinary tract dysfunction; PVR: post-void residual

Systems	Manifestations	Diagnostic tools
GI	Dysphagia	VFSS or FEES
	EMD: esophageal spasm, slow esophageal transit, aperistalsis, gastroesophageal reflux, motor disorders of the lower esophageal sphincter, and achalasia	EGD
	Gastroparesis	Gastric emptying scintigraphy
	Constipation (differentials include diverticulitis, appendicitis, incomplete evacuation and bowel incontinence, megacolon, colon obstruction, pseudo-obstruction, volvulus, perforation, and other intra-abdominal processes)	DRE and CT scan of the abdomen and pelvis
	Fecal incontinence	
GU	NLUTD and complicated UTI (fever or not responding to appropriate therapy remains warning signs of upper urinary tracts for sources of UTI, including hydronephrosis, pyonephrosis, urolithiasis, and/or renal abscess)	Urinary analysis and culture, ultrasonography or CT scan, PVR and cytology
CV	Orthostatic hypotension	Orthostatic vital signs
	Resting tachycardia	
	Exercise intolerance	
	Silent myocardial ischemia	

## Conclusions

Dysautonomia-linked comorbidities can be debilitating and sometimes fatal. Our patient was on a clinical decline majorly due to dysautonomia and nearing the end of life over the past year. A holistic approach of possible de-escalating care and palliative care might have led to a better quality of life for this patient and his family. Nevertheless, in general, emergent presentations of dysautonomia symptoms in patients with PD should be recognized and treated timely and appropriately in the emergency room. Emergency clinicians need to increase awareness and make efforts to manage these acute worsening episodes of dysautonomia disorders in patients with PD to prevent debilitating and fatal complications.
